# Visualization of solute diffusion into cell walls in solution-impregnated wood under varying relative humidity using time-of-flight secondary ion mass spectrometry

**DOI:** 10.1038/s41598-018-28230-2

**Published:** 2018-06-29

**Authors:** Peiming Zheng, Dan Aoki, Masako Seki, Tsunehisa Miki, Soichi Tanaka, Kozo Kanayama, Yasuyuki Matsushita, Kazuhiko Fukushima

**Affiliations:** 1 Structural Materials Research Institute, National Institute of Advanced Industrial Science and Technology, Nagoya, 463-8560 Japan; 2 Graduate School of Bioagricultural Sciences, Nagoya University, Nagoya, 464-8601 Japan; 3 Research Institute for Sustainable Humanosphere, Kyoto University, Uji, 611-0011 Japan

## Abstract

The purpose of the present study is to clarify the diffusion of non-volatile substances into cell walls during the conditioning procedure under varying relative humidities (RH). In this paper, wood blocks were impregnated using an aqueous solution of melamine formaldehyde (MF), and they were subsequently conditioned under RHs of 11, 43, and 75%. The solute that diffused into the cell walls was visualized using time-of-flight secondary ion mass spectrometry (TOF-SIMS). The volumetric relative swelling of the samples during the conditioning procedure was calculated. The results showed increased cell wall swelling at higher RH, which may have been caused by higher MF diffusion into the cell walls and/or higher moisture content. Cryo-TOF-SIMS measurements showed that more cell cavities were unfilled with MF at higher RH, indicating that most of the MF diffused from the cell cavities into the cell walls. The relative intensity of MF in the cell walls of the cured samples was evaluated from dry-TOF-SIMS images, which showed a higher relative intensity of MF in the cell walls at higher RH. With the ability to visualize and semi-quantitatively evaluate the solute in cell walls, TOF-SIMS will serve as a powerful tool for future studies of solute diffusion mechanisms in solution-impregnated wood.

## Introduction

Treatment using non-volatile substances is an effective method for reducing the undesirable properties of wood materials, such as “non-homogeneity, dimensional instability in moist environments, and susceptibility to sunlight, fungi, and insects”^1,2^. Wood properties (e.g., mechanical properties and dimensional stability) have been significantly improved by treatment with appropriate substances in previous studies^3–6^. It has been shown that depositing solute within wood cell walls rather than in cell cavities results in a high decay resistance of the wood as well as an improvement in its mechanical strength and dimensional stability^6–9^. Thus, maximizing the solute amount in cell walls is important for ensuring treatment efficiency^2,6^.

Treatment of wood with non-volatile substances includes impregnation and conditioning^2,6^. The mechanism of solute diffusion into the cell wall has been suggested by Stamm^10^ and Tanaka *et al*.^2,6^: “(i) During impregnation, the solution permeates the cell walls and cell cavities uniformly. (ii) During conditioning, a greater amount of solvent evaporates from the cell cavities compared with that from the cell walls, which results in higher concentration of solute in the cavities compared with that in the walls. This concentration difference causes the diffusion of the solute from the cavities to the walls.” It was concluded that the amount of solute diffused into the cell wall is determined by two factors, the difference in solute concentration between the cell cavities and walls and solute diffusivity into the cell walls. Tanaka *et al*.^2^ concluded that both factors are affected by “solution conditions (such as the concentration and type of solute and solvent), material conditions (such as wood species and dimension), and atmospheric conditions (such as temperature and vapor pressure of the solvent)”. Optimizing the atmospheric conditions is more feasible, because the other conditions often depend on the required properties of the treated materials^2,11^. Therefore, there exists the possibility of maximizing the solute amount in cell walls by optimizing the vapor pressure of the solvent [i.e., the relative humidity (RH) of the aqueous solution].

To investigate the diffusion of solute into the cell wall, it is necessary to choose an appropriate method, which measures its amount and distribution in the cell walls. Tanaka *et al*.^6^ estimated the solute in cell wall by the dimensions of the solution-impregnated wood with polyethylene glycol (PEG). Images from micro-focus X-ray computed tomography (CT) were also used to investigate the resin polymer distribution in cell cavities^2^. However, the resin distribution in the cell walls could not be evaluated. Furuno *et al*.^3^ investigated the penetration of solute into wood cell walls using electron probe X-ray microanalysis (EPMA) and a m-Bromophenol-formaldehyde resin was used to detect the presence of resin by bromine signals. However, the drying procedure was conducted on samples in these studies. Although the amount of solute diffusing into the cell walls was considered to be small during the drying procedure^2,12^, drying and curing procedures lead to shrinkage of samples and/or condensation of the solute [e.g., while forming methylene linkages during curing of melamine formaldehyde (MF)]^13^. Thus, solute diffusion into the cell wall affected by RH during the conditioning procedure could not be directly measured.

In the present study, time-of-flight secondary ion mass spectrometry (TOF-SIMS) was employed to map the solute distribution in the cell walls of impregnated wood. TOF-SIMS is an analytical technique having high mass and spatial resolution, and provides semi-quantitative information on the chemical features of the surface of untreated solid samples^14–19^. TOF-SIMS has been successfully used to visualize the solute distribution in solution-impregnated wood^20,21^. The sample preparation process is quite important to visualize the actual distribution of the target chemicals^22^. To avoid movement, draining, or change of the water-soluble chemicals during additional drying process, cryo-TOF-SIMS with frozen samples was recently developed^15,23,24^.

In the present study, MF was used as the solute in the impregnation treatment. It was reported that, “as one of the hardest and stiffest isotropic thermosetting polymeric materials, MF provides various material advantages for improving wood properties, such as transparency, thermal stability, scratch resistance, moisture resistance, and surface smoothness”^13,25^. The purpose of this study is to visualize and evaluate MF diffused into the cell walls of wood after conditioning at various RHs. Weight percent gain (WPG) and volumetric swelling behavior of the wood impregnated with an aqueous solution of MF were evaluated during conditioning at various RHs. The MF distribution in freeze-fixed samples after conditioning was visualized using cryo-TOF-SIMS. The conditioned wood was subsequently cured and a cross section was investigated using dry-TOF-SIMS.

## Results and Discussion

### WPG and relative swelling during conditioning

**Figure 1:**
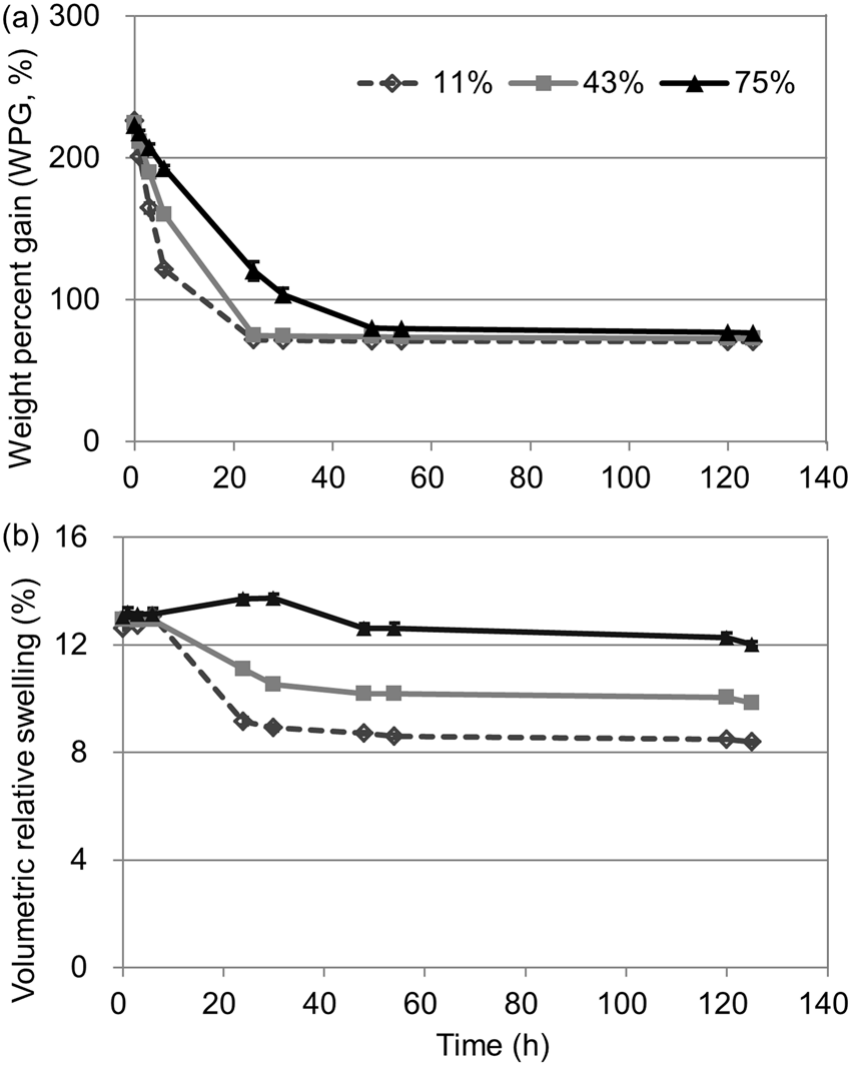
Temporal variability of (**a**) weight percent gain and (**b**) volumetric relative swelling of the MF-impregnated samples conditioned at various RHs (11, 43, and 75%). The bars show the standard deviation from three replicates.

Figure 1a shows the temporal variability of WPG during conditioning at various RHs. At the start of the conditioning, which was 50 h before (Fig. 1a), the WPG decreased indicating water evaporation. Additionally, higher WPG was observed at higher RH, which is consistent with previous studies using PEG as the solute^2,6^. This finding attributes to higher RH (higher vapor pressure) leading to slower water evaporation.

After 50 h of conditioning, the value of WPG at different RHs was similar and had reached equilibrium (Fig. 1a). However, the value of WPG after vacuum drying was significantly different (*P* < 0.01), i.e., 69.45% ± 0.26%, 66.64% ± 0.08%, and 61.02% ± 0.26% for RHs of 11, 43 and 75%, respectively. These results indicate that MF itself degenerated during the conditioning and that the degeneration degree was RH dependent. Previous studies have revealed that polymerization can occur during the conditioning procedure for some resins because of dehydration, such as phenol formaldehyde resin^26–28^. Additionally, higher RH can promote degradation^26,27^. Similar polymerization might have occurred for MF in the present study, because MF polymerization also occurs as a result of dehydration^13^. The relative moisture content of the samples after conditioning, as calculated from the weight of the vacuum-dried samples, was significantly different (*P* < 0.01), i.e., 1.24% ± 0.03%, 6.23% ± 0.03%, and 15.76% ± 0.32% for RHs of 11, 43 and 75%, respectively, which is consistent with previous studies using PEG as the solute^2^.

Figure 1b shows the temporal variability of the volumetric relative swelling during the conditioning procedure at various RHs. Similar with temporal variability of the WPG (Fig. 1a), the relative swelling values decreased at the start of conditioning (Fig. 1b). As reported by previous research^2^, the volumetric relative swelling values shown in Fig. 1b should be multiple results of swelling caused by MF diffusing into the cell walls and shrinkage caused by water exuding from the cell walls. A slower decrease in relative swelling was observed at higher RH at the start of conditioning (Fig. 1b), which is attributed to higher solute diffusivity and/or slower water exudation.

After 50 h, the relative swelling reached equilibrium and the samples at higher RH exhibited higher relative swelling (Fig. 1c). It is possible that the higher relative swelling was caused by the larger amount of MF diffusing into the cell walls at higher RH. On the other hand, as mentioned above, the samples at higher RH had a higher moisture content, which contributed to the cell wall swelling. Therefore, MF diffusion into the cell walls cannot be verified by the WPG and volumetric relative swelling results only.

### Positive ion TOF-SIMS spectra

**Figure 2:**
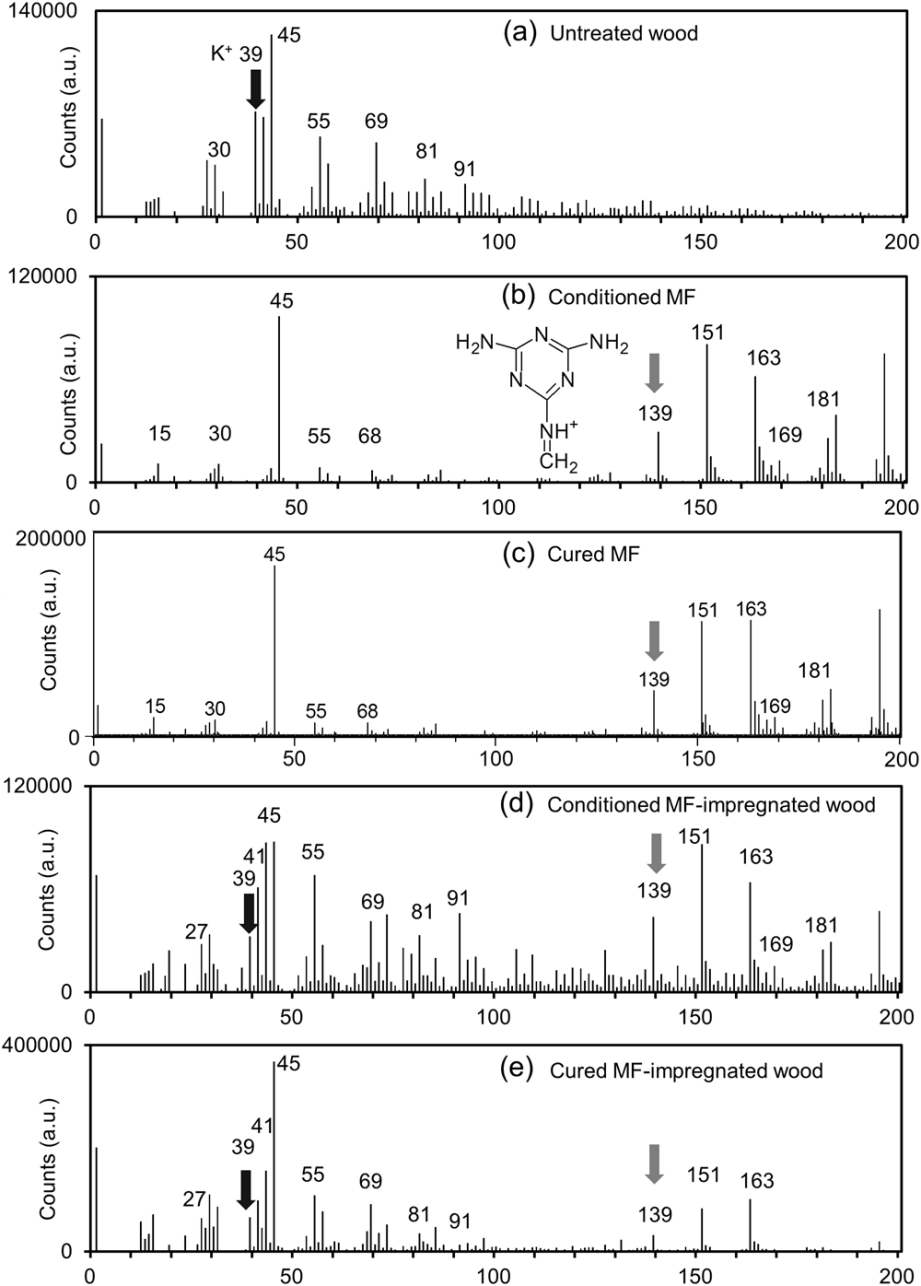
Positive ion TOF-SIMS spectra obtained from (**a**) untreated wood, (**b**) conditioned MF at RH of 43%, (**c**) cured MF, (**d**) conditioned MF-impregnated wood at RH of 43%, and (**e**) cured MF-impregnated wood. The characteristic peaks of K^+^ at *m/z* 39 and the fragment MF ion at *m/z* 139 are shown. The structure of the fragment obtained from MF at *m/z* 139 is also inset in (**b**).

The typical positive ion spectra obtained from the untreated wood, conditioned MF at RH of 43%, cured MF, conditioned MF-impregnated wood at RH of 43%, and cured MF-impregnated wood in the mass region *m/z* of 0–200 are shown in Fig. 2a–e, respectively. Coullerez *et al*.^29^ reported that *m/z* 139 ([C_4_H_7_N_6_]^+^), 151 ([C_5_H_7_N_6_]^+^), 163 ([C_6_H_7_N_6_]^+^), and 181 ([C_6_H_9_N_6_O]^+^) are the main characteristic peaks for both uncured and cured MF in a TOF-SIMS measurement. However, it was reported that *m/z* 151 ([C_8_H_7_O_3_]^+^ and [C_9_H_11_O_2_]^+^), 163 (H^+^ (H_2_O)_n_), and 181 (H^+^ (H_2_O)_n_ or [C_9_H_9_O_4_]^+^ and [C_10_H_13_O_3_]^+^) are also typical for lignins or water cluster ions in wood samples^15–17,30^, which overlap with the MF characteristic peaks. Thus, the peak of *m/z* 139 ([C_4_H_7_N_6_]^+^) in the MF-impregnated wood samples investigated here is assigned as the characteristic peak of MF.

On the other hand, the signal *m/z* 39 is assigned to K^+^, which is mainly from wood cell walls. Moreover, inorganic K^+^ (exact *m/z* 38.96) is distinguishable from organic C_3_H_3_ (exact *m/z* 39.02) in image mode as shown in our previous study^15^. Therefore, the peak of *m/z* 39 (K^+^) was used to map the wood cell walls as reported in many previous studies^23,24,33^.

### MF distribution in conditioned MF-impregnated wood by cryo-TOF-SIMS

**Figure 3:**
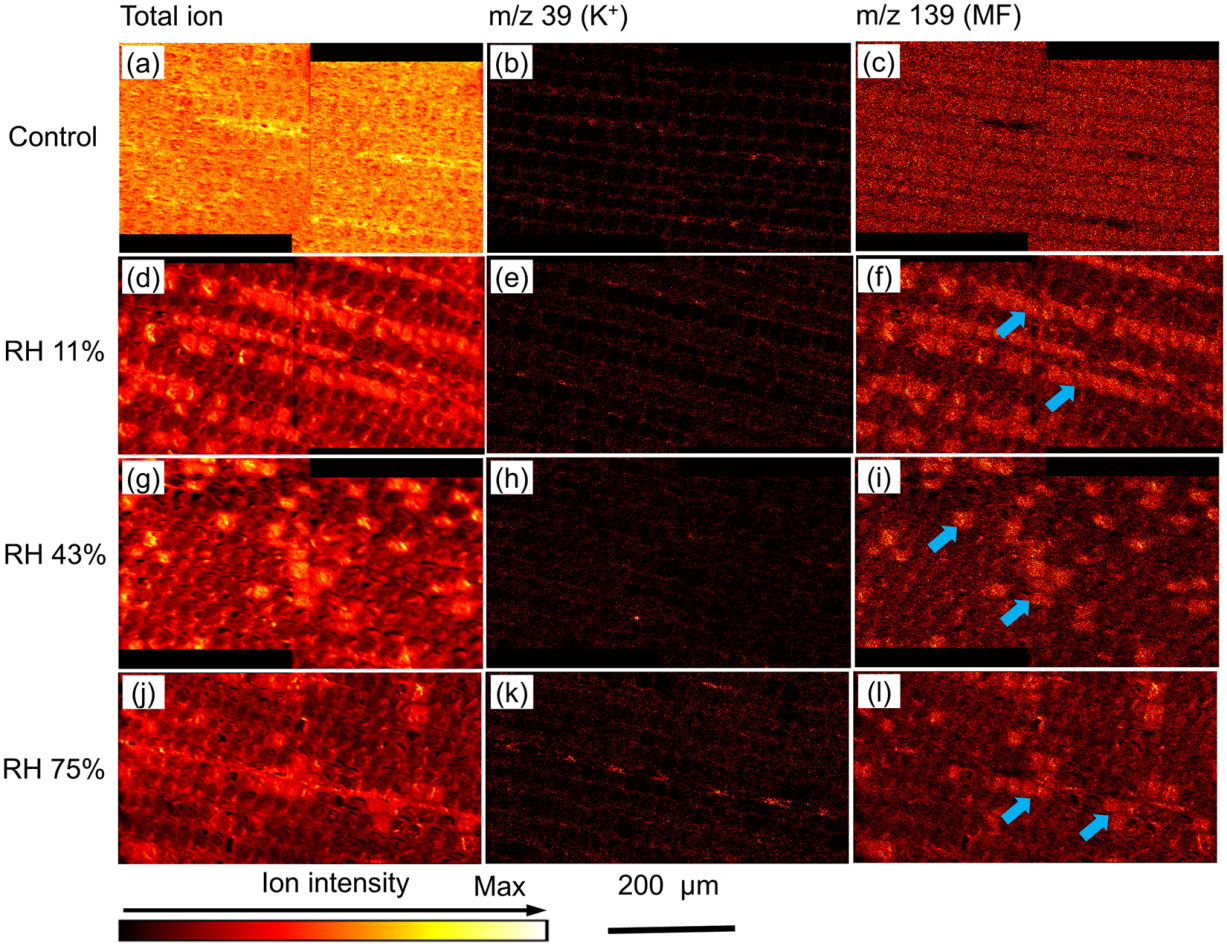
Cryo-TOF-SIMS images of the freeze-fixed MF-impregnated wood without conditioning (**a**–**c**; control), and with conditioning at RH of 11% (**d**–**f**), 43% (**g**–**i**), and 75% (**j**–**l**). The images of the total ion, *m/z* 39 (K^+^), and *m/z* 139 (MF) are shown. Arrows indicate the cell cavities filled with MF.

Representative cryo-TOF-SIMS images of the freeze-fixed MF-impregnated wood without conditioning (Figures a–c; control) or with conditioning at a RH of 11% (Figures d–f), 43% (Figures g–i), and 75% (Figures j–l) are shown in Fig. 3. The images of the total ions, *m/z* 39 (K^+^), and *m/z* 139 (MF) are arranged on the left, middle, and right side, respectively (Fig. 3). The intracellular regions were filled by MF and/or water (as shown in Fig. 3a,d,g and j). Although the cell walls were clearly illustrated by mapping K^+^ in the control sample (Fig. 3b), K^+^ diffused into the cell cavity with the conditioning process (Fig. 3e,h and k).

In the MF-impregnated wood without conditioning, almost all the cell cavities were filled with the MF solution (Fig. 3c). The relative intensity of MF in the cell cavities was higher than that in the cell walls. This is because in comparison to the cell walls, the cell cavities can be more easily permeable to the MF solution in the impregnation process.

**Figure 4:**
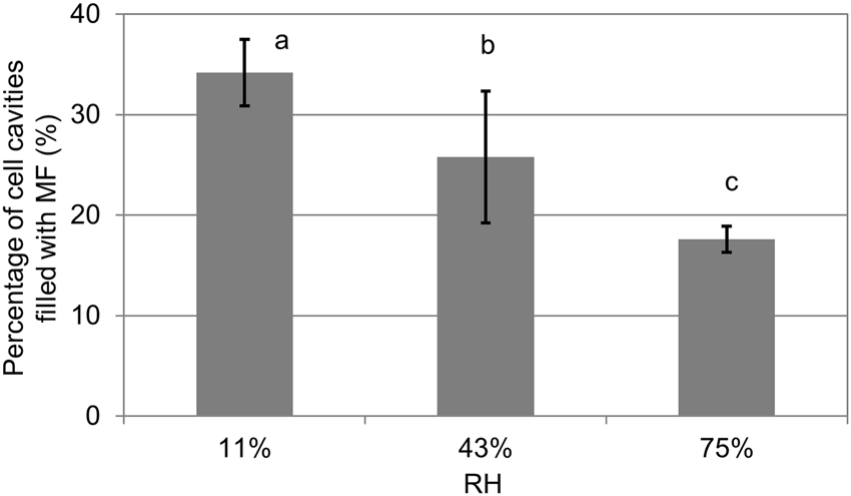
Percentage of cell cavities filled with MF as evaluated from the cryo-TOF-SIMS images in Fig. 3. The bars indicate the standard deviation from five replicates. Different letters indicate significant differences (*P* < 0.01).

During the conditioning procedure at RH of 11%, a number of lines of cell cavities were filled with MF (Fig. 3f, arrows). With increase in RH, the numbers of cell cavities filled with MF decreased (Fig. 3i and l, arrows). Figure 4 shows the percentage of cell cavities filled with MF as calculated from the cryo-TOF-SIMS images (Fig. 3). It shows that a significantly lower percentage of cell cavities were filled with MF at higher RH (Fig. 4, *P* < 0.01), which indicates higher amounts of MF diffusing into the cell walls. These results are consistent with previous studies^2,11^.

Previous studies reported that the amount of solute diffusion into the cell wall is determined by the difference of solute concentration between the walls and the cell cavities and solute diffusivity into the cell walls^2,6,10^. At lower RH, the higher evaporation rate of water results in a larger concentration difference between the cell walls and the cell cavities. However, lower RH reduces the solute diffusivity into the cell walls^2,11^. From the results of the present study, it can be concluded that the RH effect on solute diffusivity is greater than that on concentration difference between the cell walls and cell cavities during the conditioning process. Therefore, optimizing the RH to find a balance between the two factors (i.e. solute diffusivity and concentration difference between cell walls and cell cavities) is necessary for ensuring treatment efficiency.

### MF distribution in cured MF-impregnated wood by dry-TOF-SIMS

**Figure 5:**
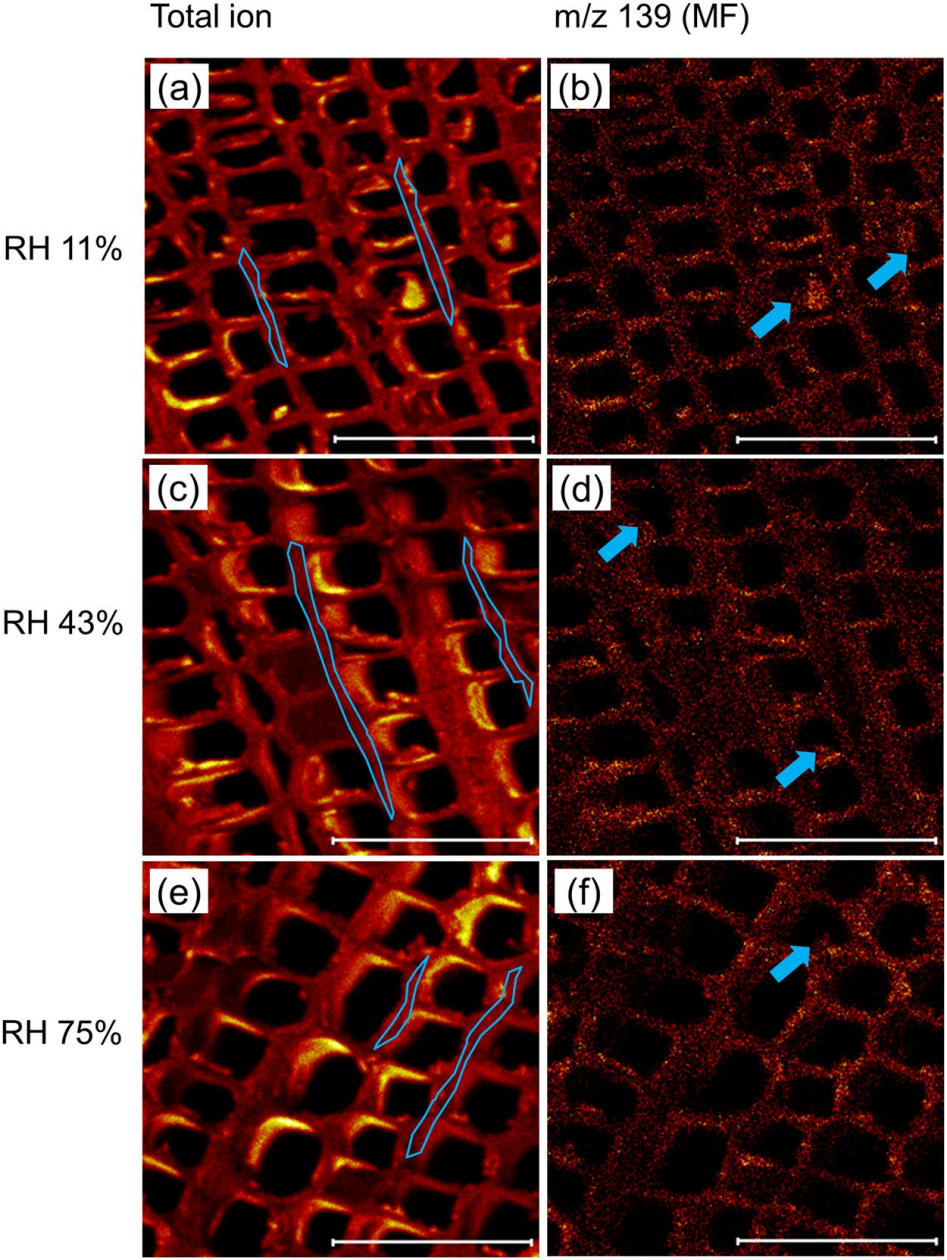
Representative total ion images and MF distribution maps of the cured samples conditioned under RH of 11% (**a** and **b**), 43% (**c** and **d**), and 75% (**e** and **f**). The typical ROIs of the cell walls are enclosed with blue lines. Arrows indicate MF deposited on the inner surface of the cell walls. Scale bar: 100 μm.

The total ion and *m/z* 139 of MF are clearly mapped in Fig. 5. From the results, it is evident that MF was mainly located in the cell walls (Fig. 5b,d and f). The MF solution in the cell cavities was reduced because of water evaporation and MF polymerization, and deposited on the inner surface of the cell wall during drying and curing processes (Fig. 5b,d and f, arrows). It was difficult to evaluate the MF amount differences among various RHs only by checking the brightness of the images.

**Figure 6:**
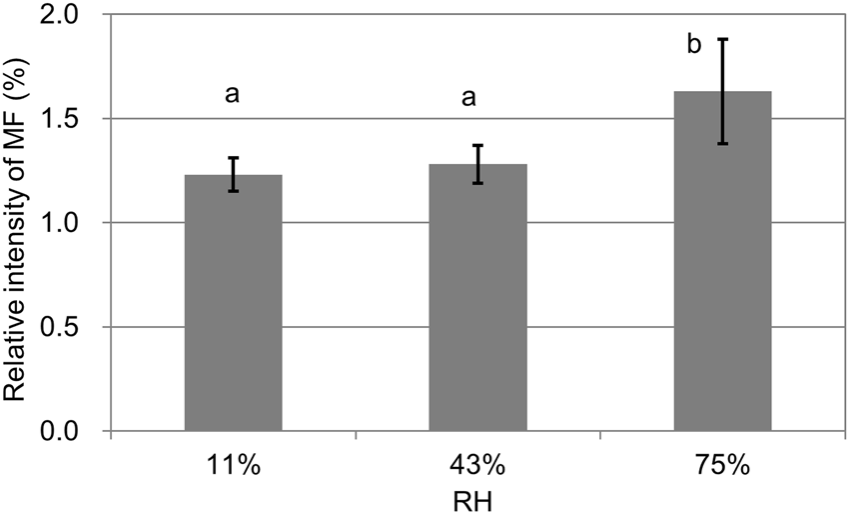
Relative intensity of MF in the cell wall of the cured samples as shown in Fig. 5. The bars represent standard deviation from five replicates. Different letters indicate significant differences (*P* < 0.01).

Thus, region-of-interest (ROI) function of TOF-SIMS was employed. Typical ROIs of the cell walls are enclosed with blue lines in Fig. 5a,c and e. The relative amount of MF-related ions was well quantified based on the total ions (Fig. 6), i.e., by dividing the MF (m/z 139) ion counts by the total ion counts. Figure 6 illustrates that the relative intensity of MF in the cell walls at RH of 75% was significantly higher than that at RH of 11 and 43% (*P* < 0.01). This means more MF diffused from cell cavity into cell wall at RH of 75%, which is consistent with less MF in cell cavity of the cryo-TOF-SIMS results (Figs 3 and 4). On the other hand, there is insignificant difference between the results at RH of 11 and 43% (Fig. 6). In Fig. 4, the standard deviation is large at RH of 43%, which indicates that the MF distribution is uneven between the different regions. This may have caused the insignificant difference between the results at RH of 11 and 43% in Fig. 6. Additionally, the limitations of TOF-SIMS due to ionization efficiency and the so-called matrix effect may be another factor^16,17,31^.

**Figure 7:**
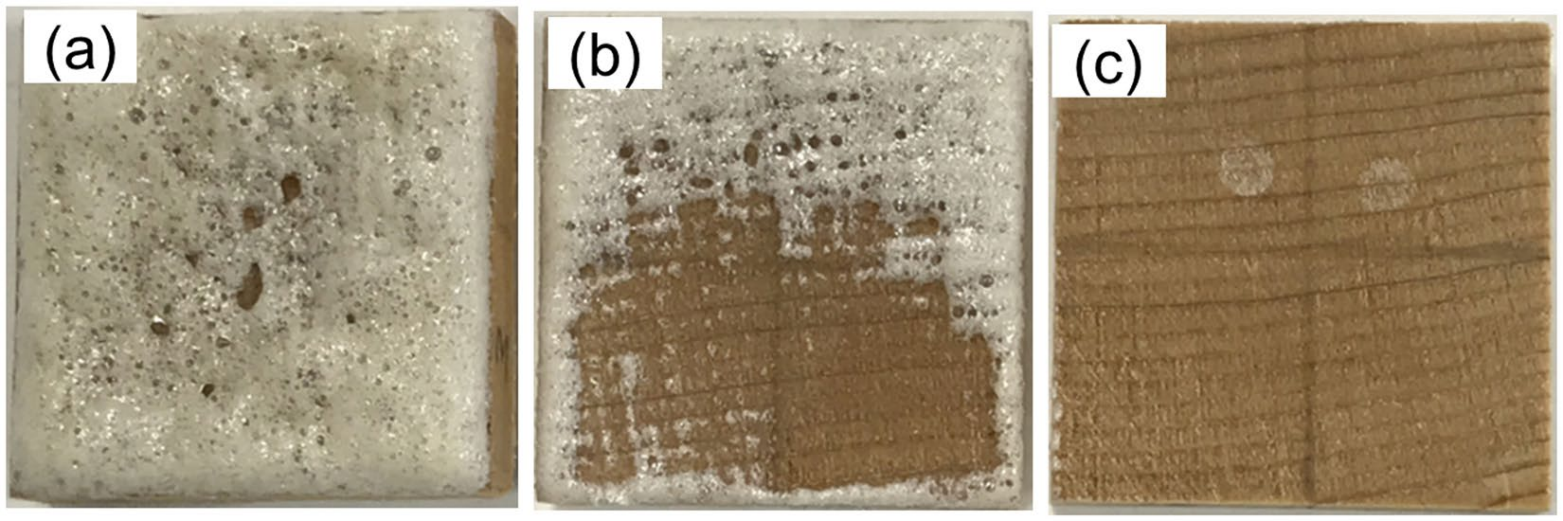
Representative photographs of the MF-impregnated wood after conditioned at RH of 11% (**a**), 43% (**b**), and 75% (**c**), and subsequently vacuum dried.

From the representative photographs of MF-impregnated wood after conditioning at various RHs and subsequent vacuum drying (shown in Fig. 7), it is evident that there was a larger amount of MF exuding from the sample surface accompanied by bubbles at lower RH. The MF in the cell cavities can exude from the sample surface under vacuum condition. In another hand, MF in the cell walls fills the amorphous regions^6^ and is polymerized by dehydration^13,26–28^, which is difficult to exude from the sample surface. Therefore, this result shows that there was a larger amount of MF in the cell cavities rather than the cell walls at lower RH, which confirms the TOF-SIMS results (Figs 3–6).

## Conclusions

The MF distribution of impregnated and subsequently conditioned wood under various RHs was visualized using cryo- and dry-TOF-SIMS. Measurement of the dimensional variability showed a larger volumetric relative swelling of the cell walls at higher RH. Cryo-TOF-SIMS showed that more cell cavities remained unfilled by MF at higher RH, which indicates more MF diffusion into the cell walls. The relative intensity of MF in the cell walls was evaluated by dry-TOF-SIMS and the results showed that there was a larger amount of MF in the cell walls at higher RH.

TOF-SIMS has the ability to detect molecular or fragment ions of MF and subsequently map their distribution on the solution-impregnated sample surface with microscopic lateral resolution. A semi-quantitative evaluation of MF in the cell walls can be performed. TOF-SIMS will be a powerful tool for future studies of solute diffusion mechanisms in solution-impregnated wood.

## Methods

### Sample treatment

Wood samples were prepared from a block of *Chamaecyparis obtusa* with nominal dimensions of 5 mm × 25 mm × 25 mm in the longitudinal, radial, and tangential directions, respectively. The samples were dried at 105 °C to a constant mass, followed by measurement of their weights (W_0_) and dimensions (V_0_). The density of the dry samples was uniform (at 0.41 ± 0.002 g cm^−3^).

The oven-dried samples were soaked in a 30% aqueous solution of MF [Beckamine M−3 (60), DIC Corporation, Osaka, Japan; average molecular weight: approximately 350] under vacuum at a pressure of 0.01 MPa for 2 h and subsequently subjected to a pressure at 0.8 MPa for 18 h in an impregnation plant (YA-10, Yasujima, Kanazawa, Japan). The impregnated samples were blotted and their weights and dimensions were measured.

The samples were divided equally into four groups. One group was immediately frozen in liquid Freon R22 (DuPont) at −160 °C without conditioning and stored at −80 °C. The other groups were conditioned for 125 h in a desiccator (MD-1, Sanplatec, Osaka, Japan) at RHs of 11, 43, or 75% (at 35 °C) using supersaturated solutions of lithium chloride (LiCl), potassium carbonate (K_2_CO_3_), and sodium chloride (NaCl), respectively^32^. The weights (W_i_) and dimensions (V_i_) of the samples were measured frequently during the conditioning procedure. The WPG and the volumetric relative swelling of the samples during conditioning procedures were then calculated using the equations 1 and 2 respectively:

1 $${\rm{WPG}}=({{\rm{W}}}_{{\rm{i}}}-{{\rm{W}}}_{{\rm{0}}})/{{\rm{W}}}_{0}\times 100 \% $$

2 $${\rm{Relative}}\,{\rm{swelling}}=({{\rm{V}}}_{{\rm{i}}}-{{\rm{V}}}_{{\rm{0}}})/{{\rm{V}}}_{{\rm{0}}}\times 100 \% $$

Half of the conditioned samples were immediately frozen in liquid Freon R22 (DuPont) at −160 °C and stored at −80 °C. The other half were dried in a vacuum chamber (at 0.01 MPa and 35 °C) for 48 h and cured (at atmospheric pressure and 170 °C) for 1 h. The frozen and cured samples were prepared for cryo- and dry-TOF-SIMS (ULVAC-PHI, Inc., Kanagawa, Japan), respectively.

### Cryo-TOF-SIMS measurement

Four kinds of frozen sample blocks (i.e., the impregnated samples and the conditioned samples at RHs of 11, 43, and 75%) were cut and set to the sample holder with ice. Then a clean and even cross surface of the samples was cut in a glove box, where the temperature was controlled at −30 °C by cooled nitrogen gas to prevent water sublimation. Subsequently, the samples were transferred to the cryo-TOF-SIMS for measurement. Details of the cryo-TOF-SIMS system have been reported by Kuroda *et al*.^33^ and Masumi *et al*.^34^.

During the cryo-TOF-SIMS measurement, the samples were maintained at lower than −120 °C. Positive ion spectra were collected under the following conditions: primary ion, 22 keV Au_1_^+^ at a current of 5 nA; pulse width, 13 ns for unbunched image analysis, and 1.8 ns for bunched spectrum analysis. A low-energy pulsed electron gun (30.0 eV) was used for surface charge compensation^15^. The measured surface areas were 300 × 300 μm^2^ and the total ion counts were approximately 8 × 10^6^ with an acquisition time of 10 min for each image.

### Dry-TOF-SIMS measurement

The measurement conditions of dry-TOF-SIMS were similar to that of cryo-TOF-SIMS. The measured surface areas were 200 × 200 μm^2^ and the total ion counts were approximately 3 × 10^6^ with an acquisition time of 10 min for each image. The ROIs of the cell walls were selected to evaluate the relative intensity of MF.

### Statistical analysis

Significance of the differences between the samples conditioned under various RHs was evaluated by a Statistical Product and Service Solutions (SPSS 24.0, IBM), which included analysis of one-way ANOVA followed by Tukey tests at the 99% confidence level.

### Data availability

All data generated or analysed during this study are included in this published article.
